# Evaluation of newly-developed glycated hemoglobin clinical analytic reagents and chromatography column on Tosoh HLC-723 G8 Analyzer

**DOI:** 10.1016/j.plabm.2023.e00338

**Published:** 2023-10-05

**Authors:** Bo Yuan, Wei Yang, Na Zhang, Hongyan Shi, Shuangpeng Dong

**Affiliations:** a Department of Biochemistry, Tianjin Quality Control and Inspection Center for Medical Device, No.5, Haitai Huake Street, Xiqing District, Tianjin, 300384, China; b The Center of Tianjin Medical Device Evaluation and Inspection, Tianjin Administration and Market Regulation, No. 237, Hongqi South Road, Nankai District, Tianjin, 300191, China

**Keywords:** HPLC, HbA1c, Precision, Linearity, Carryover

## Abstract

**Objective:**

To evaluate the performance of newly developed glycated hemoglobin (HbA1c) clinical analytic reagents and HPLC columns, applied on Tosoh HLC-723 G8 Analyzer.

**Methods:**

Newly developed reagents and columns were used on a Tosoh HLC-723 G8 Analyzer (standard mode) system to measure both of qulity contorls and the clinical blood samples to evaluate the performances of these newly developed prodcuts including precision, accuracy, linearity, carryover, bias evaluation, correlation with commercial reagents, and stability according to CLSI recommendations.

**Results:**

The *CV* of intra-assay precision and inter-assay precision of quality control and clinical blood sample assays using Lirimax products were both less than 3.00%. And the REs of accuracy were less than 6.00%. Linearity: R^2^ = 0.9993 in the concentration range 4.77%–14.67%. Carryover: 0.05%. The Bland-Altman mean difference: −0.003583% HbA1c (CI: 0.07398: −0.08115); Passing-Bablok regression: y = 1.0022(0.9984:1.006)x-0.01097(-0.03776: 0.01582), R^2^ = 0.9996. Stability evaluation was also acceptable.

**Conclusion:**

The performance of newly developed products was well evaluated for HbA1c measurement on a TOSOH G8 Analyzer which shows excellent suitability for clinical assay.

## 1 Introduction

Nowadays 500∼600 million people suffered from diabetes all over the world and around 200 million people were unconscious prediabetes [[1], [2], [3], [4]]. About 10%–13% of population in China is diabetic or prediabetic because of unhealthy lifestyle and changes in diet [5,6]. If prediabetic patients were diagnosed and treated in earlier stage, their blood glucose levels could be reverted back to normal status. In addition, maintainin normal range of blood glucose in diabetic patient leads to decreased morbidity and mortality caused by diabetic complication. Therefore, the early and precise diagnosis was essential for patients with diabetes and prediabetes.

As known, HbA1c is a specific subfraction of glycated hemoglobin, which indicates average level of blood glucose concentration over 8–12 weeks, to assess long-term glycemic control and therapeutic effect as well as a predictor for the risk of developing microvascular complications of diabetes [[7], [8], [9]]. Many clinical studies and trials suggested that HbA1c levels may be associated with cardiovascular disease [10], diabetic stroke, diabetic nephropathy, chronic microvascular complications and fundus oculi lesion, *etc.* So far HbA1c has been recommended for screening and diagnosis of diabetes by American Diabetes Association [[11], [12], [13]]. Similar recommendations were proposed by WHO as well as other countries.

Until now, various methods have been developed and updated such as enzymatic assay, capillary electrophoresis, boronate affinity HPLC, ion-exchange HPLC and immunoassay, *ect.* Among them, enzymatic assay, boronate affinity HPLC and ion-exchange HPLC were popular in clinical HbA1c analysis [14]. Ion-exchange HPLC, considered as the golden standard of clinical HbA1c analysis, is the most popular method for HbA1c analysis [15,16]. Its analytical system usually includes automatic glycohemoglobin analyzer machine, elution buffer, hemolysis reagent, chromatography column, calibtrator and qulity control. The performance of machine usually cannot be modified after leaving the factory [15,[17], [18], [19]]. Except analyzer, the precise analysis still need specific elution buffer, hemolysis reagent, chromatography column, calibtrator and qulity control. Elution buffer could gradiently eluted all hemoglobin from chromatography column. Hemolysis reagent is cell lysis buffer which disrupted erythrocytes to release hemoglobin and clean equipment's pipes. Chromatography column as a hemoglobin adsorption and dissociation carrier is the core of components. Calibrator adjusts the machine to get accuracy value. And quality control is used to evaluate system working satus and performance.

Recently studies focus on comparing the performance of different Automatic Glycohemoglobin Analyzer or Hemoglobin Testing System. However, few cared the performance of reagents and columns, which were also crucial in glycohemoglobin analysis.

In this study, we conducted a comprehensive evaluation of newly developed reagents and columns for use with the Tosoh HLC-723 G8 Analyzer. Our aim was to assess whether they would be compatible with the related analyzer system and produce reliable glycohemoglobin analysis results.

## 2 Materials and methods

### 2.1 Reagents

The standard references of glycated hemoglobin (GBW09181a, GBW09182a, and GBW09183a) were obtained from Clinical Test Center of Beijing Hospital Ministry of Health in China.

The evaluated HbA1c diagnostic reagents and columns: Calibrators (XN-TSG8-SC, Lirimax, China), Quality Control (XN-QC, Lirimax, China), Elution buffer No.1 (XN-TSG8-01, Lirimax, China), Elution buffer No.2 (XN-TSG8-02, Lirimax, China), Elution buffer No.3 (XN-TSG8-03, Lirimax, China), Hemolysis reagent (XN-TSG8-04, Lirimax, China) and Chromatography Columns (XN-TSG8-TC, Lirimax,China), were obtained from Lirimax (Tianjin) Medical Technology Co., Ltd.

The HbA1c diagnostic control reagents and colums: TOSOH HbA1c Calibrator set (TOSOH Corporation, Japan), TOSOH HbA1c Control HSi (TOSOH Corporation, Japan), G8 Elution Buffer HSi No.1(S) (TOSOH Corporation, Japan), G8 Elution Buffer HSi No.2(S) (TOSOH Corporation, Japan), G8 Elution Buffer HSi No.3(S) (TOSOH Corporation, Japan), HSi Hemolysis & Wash Solution (TOSOH Corporation, Japan) and Chromatography Columns TSKgel G8 HSi (TOSOH Corporation, Japan) were purchased from TOSOH Corporation.

### 2.2 Instrumentation

HLC-723 G8 Automatic Glycohemoglobin Analyzer standard mode (TOSOH Corporation, Japan) was utilized in the whole evaluation. The method for glycohemoglobin analysis was recommended by the NGSP (National Glycohemoglobin Standardization Program) and IFCC (International Federation of Clinical Chemistry and Laboratory Medicine). The analyzer was calibrated at the beginning of the measurement, according to the manufacture's instructions using manufacturer-provided calibrators.

### 2.3 Clinical blood specimens

The clinical samples in EDTA anti-coagulant tubes were collected from Department of Clinical Laboratory, the Air Force Characteristic Medical Center of PLA, China and stored at 2–8 °C for up to 72 h.

## 3 Evaluation protocol

### 3.1 Precision

In the precision evaluation, there were two sets of testing samples: quality controls and blood specimens. The quality controls included low value product (No.Cont01, HbA1c: 5.25%) and high value product (No.Cont02, HbA1c: 9.41%). The clinical blood samples included low value (HbA1c:4.70%–5.50%, L1-L6), medium value (HbA1c:6.50%–7.50%, M1-M6) and high value (HbA1c:> 8.00%, H1–H6).

For intra-assay precision, the same lot of hemolysis reagent (only for blood samples), elution buffer and columns were utilized for 10 tests of quality control and clinical blood samples for 10 times on the calibrated Analyzer.

For inter-assay precision, the clinical blood samples and quality control were measured for 10 times with 3 lots of hemolysis reagent (only for blood samples), elution buffer and columns on the calibrated Analyzer. The mean, standard deviation and *CV* were calculated.

### 3.2 Accuracy

The standard references of glycated hemoglobins (No. GBW09181a, GBW09182a and GBW09183a) were measured for 5 times on the calibrated Analyzer, respectively. The mean, standard deviation and relative error were calculated.

### 3.3 Linearity

One low value blood sample (L, Hb1Ac<5.00%) and one high value blood sample (H, Hb1Ac>14.00%) were measured on the calibrated Analyzer. Then the samples were diluted 150-fold and mixed as indicated in Table 1. Then seven mixed samples were obtained and measured for 3 times on the calibrated Analyzer.

**Table 1:** Preparation of linear samples mixture.

No.	Volume of L (mL)	Volume of H (mL)	Total volume (mL)
1	2.0	0.0	2.0
2	1.6	0.4	2.0
3	1.2	0.8	2.0
4	1.0	1.0	2.0
5	0.8	1.2	2.0
6	0.4	1.6	2.0
7	0.0	2.0	2.0

### 3.4 Carryover

One low value Hb1Ac (HbA1c: 4.00%–6.50%, repeated injection as L1 - L5) blood sample and one high value Hb1Ac (HbA1c:≥12.00%, repeated injection as H1 – H4) sample were used in the study, then measured in the order of L1-L2-L3-L4-H1-H2-H3-H4-L5. The data were analyzed for carryover determination.

### 3.5 Bias and correlation

120 clinical blood samples were measured using the reagents and columns from both Lirimax and TOSOH, respectively.

### 3.6 Stability

For long term stability, the performance of reagents and chromatography column were tested on the 3rd,6th, and 9th month, respectively, as above-mentioned in methods.

### 3.7 Statistical analysis

Statistitics was performed using GraphPad Prism software, version 5.0 (GraphPad Software Inc, La Jolla, CA). The data were presented with means, SDs, coefficients of variation (*CV*), correlation coefficients (r).

## 4 Results

### 4.1 Precision

Both intra-assay and inter-assay precision are summarized in Table 2 and Table 3. We tested them using quality control product as well as clinical blood samples. All *CV* were below 3.00% which meets CLSI (Clinical and Laboratory Standards Institute) criteria in HbA1c analysis.

**Table 2:** Precision study of quality control.

Qulity Control	Target Value	Intra-assay precision		Inter-assay precison	
		Mean ± SD	*CV* (%)	Mean ± SD	*CV* (%)
No. Cont01	5.25	5.09 ± 0.035	0.69	5.22 ± 0.063	1.22
No.Cont02	9.41	9.43 ± 0.023	0.24	9.35 ± 0.068	0.72

**Table 3:** Precision study of clinical blood samples.

Sample (HbA1c%)	Intra-assay precision		Sample (HbA1c%)	Inter-assay precision	
	Mean ± SD	*CV*(%)		Mean ± SD	*CV* (%)
L1	4.50 ± 0.014	0.31	L4	4.98 ± 0.059	1.19
L2	4.80 ± 0.026	0.54	L5	4.94 ± 0.057	1.15
L3	5.02 ± 0.023	0.46	L6	4.91 ± 0.055	1.12
M1	7.08 ± 0.020	0.28	M4	7.74 ± 0.085	1.10
M2	6.66 ± 0.043	0.65	M5	6.69 ± 0.098	1.46
M3	7.34 ± 0.019	0.25	M6	7.05 ± 0.083	1.18
H1	10.24 ± 0.030	0.30	H4	11.39 ± 0.031	0.27
H2	13.28 ± 0.039	0.30	H5	9.43 ± 0.045	0.48
H3	8.34 ± 0.026	0.32	H6	12.84 ± 0.049	0.38

### 4.2 Accuracy

The accuracy of noval reagents and columns was evaluated by measuring 3 levels of Standard References. All of the relative errors were less than 6.00%, a common criteria of National Health Commission of China in Table 4.

**Table 4:** Accuracy study of standard references.

Standard References	Target Value	Mean ± SD	RE
GBW09181a	5.02 ± 0.15	5.01 ± 0.032	−0.12%
GBW09182a	6.86 ± 0.15	6.72 ± 0.028	−2.07%
GBW09183a	9.34 ± 0.21	9.40 ± 0.023	0.69%

### 4.3 Linearity and carryover

**Fig. 1:**
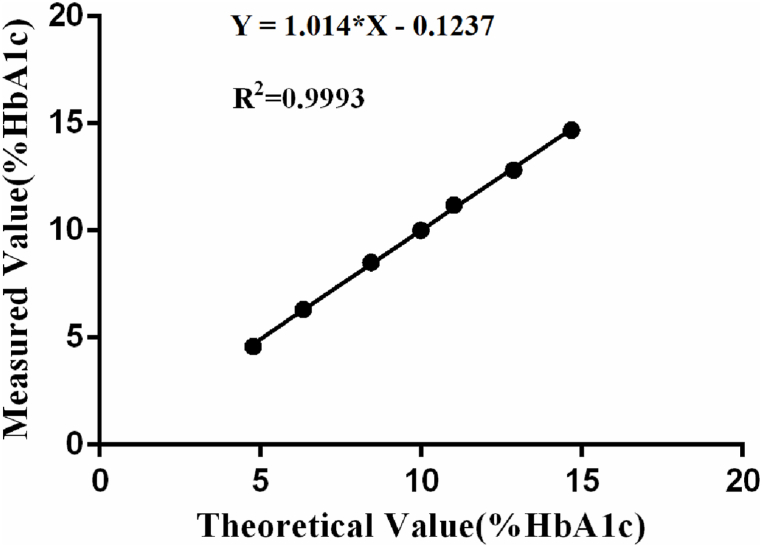
Correlation between measured values and theoretical values (target values) of HbA1c.

HbA1c levels were ranged 4.77%–14.67%. The linear regression showed measured value well matched target value (R^2^ = 0.9993) as shown in Fig. 1. The carry-over between high- and low-levels of HbA1c measured using the reagent and columns from Lirimax was 0.05%.

### 4.4 Bias and correlation

**Fig. 2:**
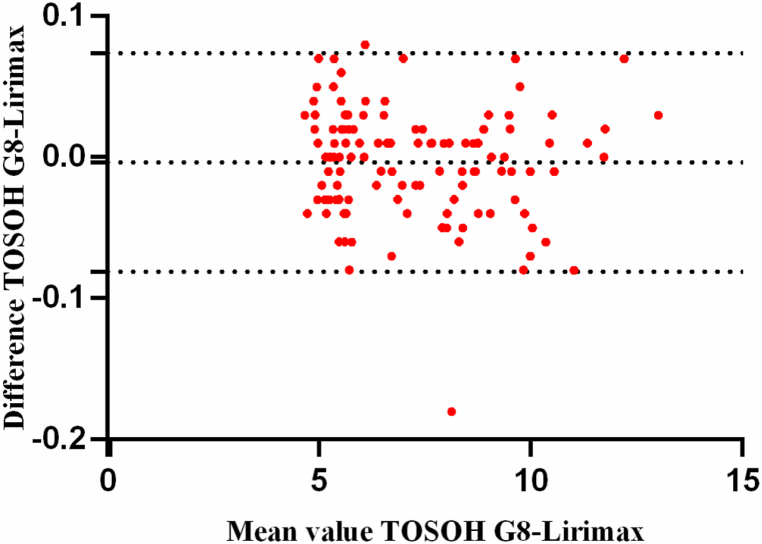
Bland-Altman difference plot: TOSOH G8 *vs* Lirimax: mean difference (TOSOH G8-Lirimax) = -0.003583% HbA1c (95% CI: 0.07398: −0.08115).

**Fig. 3:**
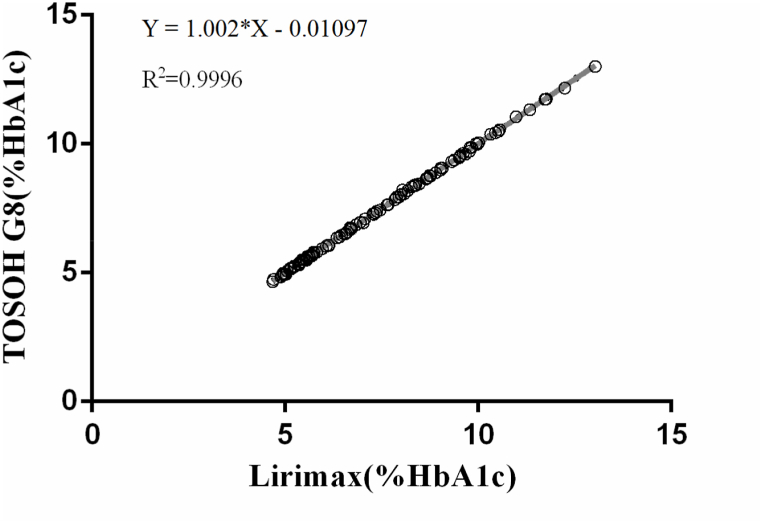
Correlation of HbA1c results measured with reagents and columns from both TOSOH G8 and Lirimax.

The results of measurements using reagents and columns from Lirimax and TOSOH G8 showed a high concordance in 120 clinical samples. The Bland-Altman plot showed a mean difference of −0.003583% in HbA1c (CI: 0.07398: −0.08115) in Fig. 2. Passing-Bablok regression between Lirimax and TOSOH G8 showed a slope of 1.0022 with a 95% CI 0.9984–1.006, and an intercept of −0.01097 with CI (−0.03776: 0.01582). The Pearson Correlation Coefficient was 0.9996 as shown in Fig. 3.

### 4.5 Stability

**Fig. 4:**
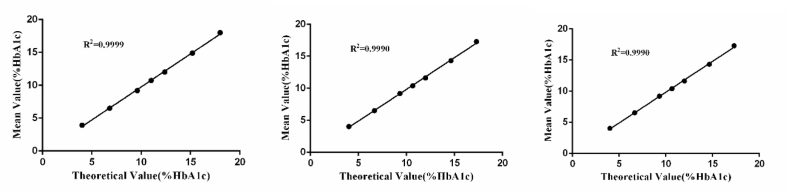
Correlation between measured values and theoretical values (target values) of HbA1c (Left, the 3rd month; Middle, the 6th month; Right, the 9th month).

The product stability was also assessed. The accuracy, precision and linearity of the products from Lirimax on the 3rd, 6th, and 9th month were evaluated. All of them were acceptable according to the common criteria. All data was shown in Table 5, Table 6 and Fig. 4.

**Table 5:** Stability study of accuracy.

Standard References	Target Value	Mean ± SD	RE (%)
		3rd 6th 9th	3rd 6th 9th
GBW09181a	5.02 ± 0.15	4.96 ± 0.055 4.90 ± 0.000 4.88 ± 0.045	−1.20-2.39-2.79
GBW09182a	6.86 ± 0.15	6.72 ± 0.045 6.76 ± 0.055 6.74 ± 0.055	−2.04-1.46-1.75
GBW09183a	9.34 ± 0.21	9.28 ± 0.045 9.26 ± 0.055 9.22 ± 0.045	−0.64-0.86-1.28

**Table 6:** Stability study of precision.

Month	Qulity Control	Target Value	Intra-assay precision	
			Mean ± SD	*CV*(%)
3rd	No. Cont01	5.25	4.97 ± 0.048	0.97
	No.Cont02	9.41	9.34 ± 0.052	0.55
6th	No. Cont01	5.25	4.97 ± 0.048	0.97
	No.Cont02	9.41	9.38 ± 0.042	0.45
9th	No. Cont01	5.25	5.05 ± 0.053	1.04
	No.Cont02	9.41	9.42 ± 0.063	0.67

## 5 Discussion

Since HbA1c was discovered in the 1960s, it has become one of the most widely used clinical biomarker of long-term blood glucose level control in prediabetic and diabetic patients. As mentioned, there are several popular methods to measure HbA1c. The guidelines for laboratory analysis of diabetes diagnosis gave a recommendation on the *CV* of intra-laboratory≤2.00% and the *CV* of inter-laboratory≤3.50% [[20], [21], [22]]. HPLC assay was recommended by various international organizations incuding IFCC [23,24].

Subquently, different instruments based on HPLC appeared and updated in the last two decades. As known, the components of HbA1c analysis system (HPLC) included the reagents such as calibrators, quality control, hemolysis reagent, elution buffer and chromatography column besides analyzer. So far there are a number of studies on evaluation of these analyzers or on the comparison of different analyzers. However, few researcher considered the evaluation of performance of reagents and column, or whether the diagnostic kit was suitable for certain analyzer. Although Analyzer plays an important role in measuring HbA1c, no doubt the performance of analytical reagents and columns is also crucial for the reproducible and unbiased measurement of HbA1c in clinical diagnosis of prediabetes and diabetes [25,26]. Therefore, we focused on the evaluation of accuracy, precision and stability of newly developped reagents and columns.

In the case of the precision, all *CV* of both intra-assay and inter-assay measuring quality control as well as clinical blood samples were below 3.00%. It indicated that these products possesed a good reproducibility under proper experimental conditions. However, precision was not enough to validate an excellent set of analytic products. It also need good accuracy to assess the difference between measured value and truth value [27]. All REs were less than 6.00%, a suggested criteria of National Health Commission of China, lower than 8.00% as claimed by TOSOH. Well-performed precision and accuracy are fundamentally important for the process of diabetes moitoring. Linearity showed that the products give reliable results within a wide range of HbA1c levels. Moreover, considering the continuously measuring by automated machine, the ture value of samples might be contaminated by adjacent samples [28]. So low carryoveris also required. The carry-over of Lirimax products was 0.05%, lower than 1.00% as claimed by TOSOH. We also compare the Lirimax reagents and column as third party products with TOSOH products and found that there was no significant difference between them in blood HbA1c analysis. Finally, we assessed the stability of these newly developed products provided by Lirimax. These products presented a stable quality for analyzing HbA1c.

In summary, the newly developed HbA1c diagnostic reagent and column exhibit excellent precision, accuracy, linearity, and minimal carryover in HbA1c measurements, as well as long-term effectiveness. These products can be used clinically as standard reagents and columns under existing standards.

## Contribution

B Y,W Y, N Z, SP D and HY S participated in the study design, analysis of the study samples, collection, analysis and interpretation of the data, B Y,W Y and SP D in writing of the report. B Y and W Y have equal contribution to the design and implementation of this study. All authors read and approved the final manuscript.

## Author statement

Bo Yuan, Wei Yang, Na Zhang, Shuangpeng Dong and Hongyan Shi participated in the study design, analysis of the study samples, collection, analysis and interpretation of the data, Bo Yuan and Wei Yang have drafted the manuscript, and Shuangpeng Dong revised it for important intellectual content. Bo Yuan and Wei Yang have equal contribution to the design and implementation of this study, all authors read and approved the final manuscript.

All persons who have made substantial contributions to the work reported in the manuscript, including those who provided editing and writing assistance but who are not authors, are named in the Acknowledgments section of the manuscript and have given their written permission to be named. If the manuscript does not include Acknowledgments, it is because the authors have not received substantial contributions from nonauthors.

## Declaration of competing interest

All authors declare that they have no competing interests for “Evaluation of Newly-Developed Glycated Hemoglobin Clinical Analytic Reagents and Chromatography column on Tosoh HLC-723 G8 Analyzer”.

## Data Availability

Data will be made available on request.

## Abbreviations

HPLC: High Performance Liquid Chromatography
CLSI: Clinical and Laboratory Standards Institute
CV: Coefficient of variation
RE: Relative error
NGSP: National Glycohemoglobin Standardization Program
IFCC: International Federation of Clinical Chemistry and Laboratory Medicine
